# Accuracy of left ventricular mechanical dyssynchrony indices for mechanical characteristics of left bundle branch block using cardiovascular magnetic resonance feature tracking

**DOI:** 10.1093/ehjci/jeae301

**Published:** 2024-11-22

**Authors:** Daniel E Loewenstein, Björn Wieslander, Einar Heiberg, Jimmy Axelsson, Igor Klem, Robin Nijveldt, Erik B Schelbert, Peder Sörensson, Andreas Sigfridsson, David G Strauss, Raymond J Kim, Brett D Atwater, Martin Ugander

**Affiliations:** Department of Clinical Physiology, Karolinska University Hospital, and Karolinska Institutet, SE-171 76 Stockholm, Sweden; Department of Clinical Physiology, Karolinska University Hospital, and Karolinska Institutet, SE-171 76 Stockholm, Sweden; Clinical Physiology, Department of Clinical Sciences Lund, Lund University, Skåne University Hospital, Lund, Sweden; Department of Clinical Physiology, Karolinska University Hospital, and Karolinska Institutet, SE-171 76 Stockholm, Sweden; Division of Cardiology, Duke University Medical Center, Durham, NC, USA; Department of Cardiology, Radboud University Medical Center, Nijmegen, The Netherlands; Allina Health Minneapolis Heart Institute at United Hospital, St. Paul, MN, USA; Department of Medicine Solna, Karolinska Institutet, and Department of Cardiology, Karolinska University Hospital, SE-171 76 Stockholm, Sweden; Department of Clinical Physiology, Karolinska University Hospital, and Karolinska Institutet, SE-171 76 Stockholm, Sweden; Division of Applied Regulatory Science, Office of Clinical Pharmacology, Office of Translational Sciences, Center for Drug Evaluation and Research, U.S. Food and Drug Administration, Silver Spring, MD, USA; Duke Cardiovascular Magnetic Resonance Center, Duke University Medical Center, Durham, NC, USA; Section of Cardiac Electrophysiology, Inova Schar Heart and Vascular, Fairfax, VA, USA; Department of Clinical Physiology, Karolinska University Hospital, and Karolinska Institutet, SE-171 76 Stockholm, Sweden; Kolling Institute, Royal North Shore Hospital, and University of Sydney, St Leonards, Sydney, NSW 2065, Australia

**Keywords:** cardiac magnetic resonance, mechanical dyssynchrony, left bundle branch block, cardiac resynchronization therapy, heart failure, circumferential strain, feature tracking

## Abstract

**Aims:**

More than 90% of patients with left bundle branch block (LBBB) and reduced left ventricular (LV) ejection fraction have LV dyssynchrony and a high probability of response to cardiac resynchronization therapy (CRT). A subgroup of patients with non-specific intraventricular conduction delay (IVCD) have a LBBB-like LV activation pattern when studied using invasive mapping and advanced echocardiographic techniques. These patients also frequently benefit from CRT, but these patients have proven difficult to identify using electrocardiogram criteria. Cardiovascular magnetic resonance (CMR) imaging indices of dyssynchrony may identify patients with IVCD who may benefit from CRT, but their relative accuracies for identification of LV dyssynchrony remain unknown. We compared the LV dyssynchrony classification accuracy of two commonly available CMR indices in a study population of patients with severely reduced LV ejection fraction and no scar and either LBBB or QRS duration <120 ms and normal QRS axis (controls).

**Methods and results:**

In LBBB (*n* = 44) and controls (*n* = 36), using CMR feature-tracking circumferential strain, dyssynchrony was quantified as the circumferential uniformity ratio estimate (CURE) and the systolic stretch index (SSI). Deidentified CMR image data were made publicly available. Both CURE and SSI quantified more severe dyssynchrony in LBBB compared with controls (P<0.001 for both). SSI more frequently discriminated LBBB and normal conduction LV activation patterns than CURE [area under the receiver-operating characteristic curve (95% confidence interval) 0.96 (0.92–1.00) for SSI vs. 0.76 (0.65–0.86) for CURE, *P* < 0.001].

**Conclusion:**

SSI is superior to CURE for discriminating synchronous and dyssynchronous LV activation and should be further studied in the setting of non-LBBB conduction abnormalities.

## Graphical Abstract

**Graphical Abstract:**
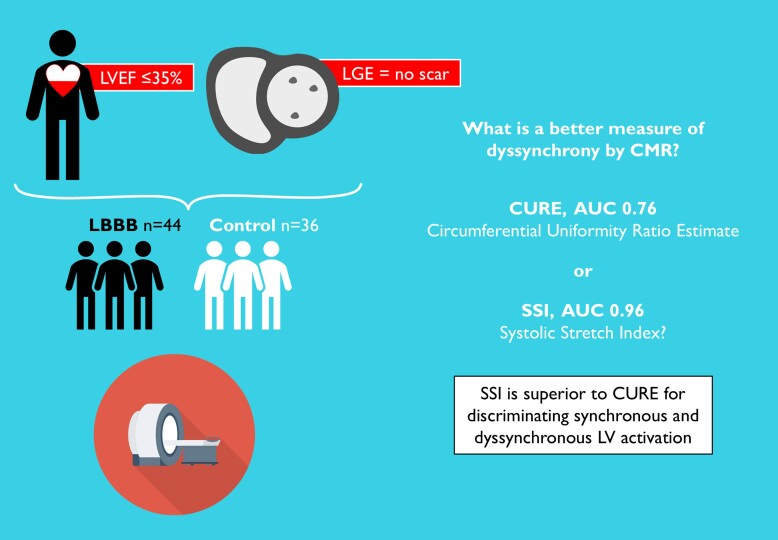
Subjects were required to have reduced LVEF ≤ 35%, no scar by CMR LGE and either normal electrocardiogram QRS duration (<120ms) and frontal plane electrical axis (−30° to +90°, controls) or LBBB defined by Strauss’ strict ECG criteria. Mechanical dyssynchrony was quantified using CURE and SSI. Abbreviations: AUC, area under the receiver-operating characteristic curve; CMR, cardiovascular magnetic resonance; CURE, circumferential uniformity ratio estimate; ECG, electrocardiogram; LBBB, left bundle branch block; LGE, late gadolinium enhancement; LVEF, left ventricular ejection fraction; SSI, systolic stretch index.

## Introduction

Cardiac resynchronization therapy (CRT) has shown major favourable effects for the treatment of patients with heart failure, severe left ventricular (LV) dysfunction, and prolonged QRS duration. The COMPANION, REVERSE, and MADIT-CRT trials demonstrated that patients with left bundle branch block (LBBB) derived greater benefit from CRT compared with those with other conduction patterns.^1–3^ Furthermore, strict LBBB, as defined by Strauss *et al*.,^4^ has, to an even greater extent, been found associated with positive CRT response and also identifies patients with a ‘super-response’ to CRT, when compared with LBBB defined by conventional criteria.^5,6^ This is likely a result of greater specificity for the strict LBBB criteria for identifying clinically significant LV dyssynchrony.

Non-specific intraventricular conduction delay (IVCD) is the second most prevalent conduction abnormality among patients receiving CRT.^7,8^ IVCD is often grouped together with right bundle branch block (RBBB), atypical LBBB, and atypical RBBB as ‘non-LBBB’. In non-LBBB, no overall benefit of CRT has previously been shown.^9,10^ Consequently, this grouping has recently been contested in a large patient-level meta-analysis including several pivotal CRT trials.^8^ In that study, patients with wide QRS (>150 ms) and either LBBB or IVCD had similar effect of CRT in reducing heart failure hospitalization or death.^8^

Considering the heterogeneity in the ‘non-LBBB’ group of patients, predicting CRT response based on QRS duration and electrocardiogram (ECG) morphology in isolation could be a too simplistic approach.^11^ Cardiovascular magnetic resonance (CMR) measures of dyssynchrony may be useful in identifying those patients without LBBB or normal conduction who have mechanical dyssynchrony that may benefit from CRT. CMR studies of regional mechanics might help identify subgroups of patients with LBBB-like characteristics. An index sensitive to the specific mechanical characteristics of strict LBBB and its associated high CRT response rate would potentially facilitate an assessment of dyssynchrony in patients without strict LBBB. Therefore, the aim of the current study was to identify such an index by comparing the discriminatory ability of two currently used methods of CMR dyssynchrony measurement to distinguish patients with strict LBBB from patients with normal conduction. We also sought to control for other myocardial abnormalities that can impact the electromechanical association by only including subjects with LV ejection fraction (LVEF) ≤ 35% and no LV myocardial scar assessed by CMR late gadolinium enhancement (LGE).

## Methods

This is an observational case-control study where patients were retrospectively identified by cross-referencing the CMR and ECG databases from three centres (Duke University Medical Center, NC, USA; Pittsburgh University Medical Center, PA, USA; and Karolinska University Hospital, Stockholm, Sweden). The study was approved by the local human subject research ethics committee at each site, and all subjects either provided written informed consent or were included following a retrospective waiver of informed consent provided by the local ethics committee.

### Subject selection

Subjects considered for inclusion in the present study had a LVEF ≤ 35%, no scar by CMR LGE, CMR cine images in a LV short-axis stack, and either ECG QRS duration (<120ms) and frontal plane electrical axis (−30° to +90°, controls, *n* = 36) or LBBB (*n* = 44) defined by Strauss’ strict ECG criteria, defined as a terminal negative deflection in lead $V_{1}$ and $V_{2}$ (QS or rS configuration), a QRS duration ≥140ms for men and ≥130ms for women, and the presence of mid-QRS notching or slurring in ≥2 of leads $V_{1},V_{2},\;V_{5},V_{6}$, I, and aVL.^4^ Subjects were excluded if they had a history of congenital heart disease, CMR evidence of myocardial storage disease, atrial fibrillation, prior open-heart surgery, or LV septal wall flattening indicative of clinically significant pulmonary hypertension. The following baseline characteristics were collected: age; sex; height; weight; body surface area; body mass index; and CMR measures of LV volumes, function, and mass. Among the patients who met the inclusion criteria (*n* = 87), patients were excluded due to having Takotsubo cardiomyopathy (*n* = 1), atrial fibrillation discovered at the time of feature-tracking analysis (*n* = 1), or missing or insufficient number of diagnostic quality CMR cine images (*n* = 5). As a result, the final study group included 80 patients.

### CMR image acquisition

All imaging was performed with clinically available scanners at the respective centres. Scanners included 3 T (Siemens Verio, Erlangen, Germany) and 1.5 T systems (Siemens Avanto, Espree, or Aera, Erlangen, Germany, or Philips Intera, Best, the Netherlands), all using ECG gating and phased array receiver coils. Typical acquisition parameters for cine images were as follows: repetition time 44 ms; echo time 1.2 ms; flip angle 60°; matrix 190 × 190; slice thickness 6 mm; and temporal resolution 24 frames/cardiac cycle. Clinical reports of cardiac viability assessment were reviewed for mention of any myocardial scar by LGE.

### Image and strain analysis

Cine CMR images exported for offline myocardial strain analysis performed by an observer using commercially available software for CMR feature tracking (Segment version 3.2 R8757, Medviso, Lund, Sweden).^12,13^ All analyses were performed blinded to ECG classification. Endocardial and epicardial borders, excluding papillary muscles and trabeculations, were manually delineated in the end-diastolic reference timeframe. The end-diastolic reference timeframe was set to the timeframe immediately following the halting of the circumferential expansion and longitudinal lengthening of the LV during late diastole as viewed in a three-chamber, long-axis, and short-axis slice. This was due to the observation that there was often a delay in the closure of the mitral valve, which otherwise has commonly been used to define end-diastole. The delineation was performed in a single mid-ventricular short-axis slice. A non-rigid elastic registration strategy was used by the software to measure myocardial strain over time. For regional strain assessment, the area encompassed by the endo- and epicardial borders was segmented into regions of interest according to six segments of the American Heart Association 17-segment model. In short-axis images, the location of regional segments was determined using an angle relative to the right ventricular anterior insertion point. Circumferential strain was evaluated from the Lagrangian strain tensor between adjacent points. Mechanical dyssynchrony was quantified using the circumferential uniformity ratio estimate (CURE)^14–16^ and the systolic stretch index (SSI).^17,18^ In short, CURE is derived from the Fourier transformation of the spatial distribution of strain from myocardial segments averaged over the number of short-axis slices. CURE is then calculated as follows:


$$\mathrm{CURE}=\frac{1}{n}\overset{n}{\underset{t=1}{\sum }}\;\sqrt{\frac{\sum S_{0}(t)}{\sum S_{0}(t)+\sum S_{1}(t)}},$$

where $S_{0}$ is the zero-order term and $S_{1}$ is the first-order term in the Fourier transformation and *n* is the number of timeframes covering the cardiac cycle. CURE ranges between 0 (perfect dyssynchrony) and 1 (perfect synchrony). SSI was originally developed through computer simulations^18^ and later presented in a slightly simplified version for use in echocardiography^17^ and is calculated as the sum of LV lateral wall systolic pre-stretch (SPS) and septal rebound stretch (SRS).


$$\mathrm{SSI}=\frac{\mathrm{SP}S_{\mathrm{antlat}}+\mathrm{SP}S_{\mathrm{inflat}}}{2}+\frac{\mathrm{SR}S_{\mathrm{antsept}}+\mathrm{SR}S_{\mathrm{infsept}}}{2}.$$

Systolic pre-stretch is defined as the sum of LV lateral wall stretch before aortic valve opening, averaged over the anterolateral and inferolateral segment. Septal rebound stretch is defined as the sum of septal stretch following early systolic shortening and before aortic valve closure, averaged over the anteroseptal and inferoseptal segments.

### Statistical analysis

Categorical data are reported as number and percentages. Continuous variables are reported as median (interquartile range). For continuous variables, groups were compared using the Wilcoxon signed-rank test. Non-parametric testing was chosen after visual inspection of the distribution of data. Bivariate correlation was examined by Spearman’s *ρ* correlation coefficient. Univariable logistic regression models with LBBB status as the dependent variable were fitted separately for the two dyssynchrony indices. Specificity, sensitivity, discriminatory performance, and cut-off values were derived from receiver-operating characteristic (ROC) curve analysis using the Youden’s index. Multivariable linear regression models fit separately for each dyssynchrony index in LBBB, and controls, respectively, were used to test for associations with covariates age, LV end-diastolic volume index (LVEDVI), LV mass index (LVMI), and sex, indicating any need for covariate-adjusted or covariate-specific ROC curves. Non-linearities were entertained by the use of restricted cubic splines, as were interactions between LVEDVI and LVMI, with sex, respectively. Areas under the paired ROC curves were compared using non-parametric stratified bootstrapping. Bootstrapped confidence intervals (CIs) were derived from 4000 replicates and calculated using the percentile method. Inter-rater and intra-rater intraclass correlation coefficient (ICC) estimates and their 95% CIs were calculated based on a single measure (*k* = 1), absolute agreement, and a two-way random-effects model, as well as a two-way fixed-effects model, respectively. Absolute reliability is reported as standard error of measurement (SEM). A two-sided P<0.05 was considered statistically significant. Data processing and statistical analysis were performed in the R statistical programming environment 4.1.0,^19^ using package dplyr 1.0.7^20^ for data transformation, ggplot2 3.3.5^21^ for graphical visualizations, pROC 1.17.0.1^22^ for ROC analysis, rms 6.2.0 for regression modelling, SimplyAgree 0.1.2^23^ for reliability analysis, and knitr 1.33^24^ for reproducible documentation.

## Results

### Subject characteristics

The characteristics of patients included in the study (*n* = 80, 56% female) are presented in *Table 1*. Characteristics were similar in the two groups except for older age and greater LV mass in subjects with LBBB.

**Table 1:** Descriptive statistics of study population

	Control *n = 36*	LBBB *n = 44*	*P* value
Age, years	48 [36–60]	64 [59–69]	< 0.001
Male sex, *n* (%)	16 (44)	19 (43)	0.91
Height, cm	169 [160–177]	169 [157–177]	0.55
Weight, kg	85 [72–95]	79.5 [64–89]	0.21
BMI, kg/m^2^	27 [25–34]	27 [24–29]	0.28
BSA, m^2^	1.9 [1.8–2.1]	1.9 [1.6–2.1]	0.31
LEDV, mL	254 [164–307]	255 [210–291]	0.72
LEDVI, mL/m^2^	123 [101–150]	127 [114–154]	0.30
LVEF, %	25 [21–30]	27 [24–32]	0.43
LVM, g	183 [156–223]	159 [131–184]	0.001
LVMI, g/m^2^	100 [82–117]	85 [67–96]	0.001
QRS duration, ms	90 [84–101]	158 [150–170]	0.001

Data are presented as median [interquartile range] or numerator (percentage).

Tests used are as follows: Wilcoxon signed-rank test and Pearson test.

BMI, body mass index; BSA, body surface area; LVEDV, left ventricular end-diastolic volume; LVEDVI, left ventricular end-diastolic volume index; LVEF, left ventricular ejection fraction; LVM, left ventricular mass; LVMI, left ventricular mass index.

### Dyssynchrony measurements

**Figure 1:**
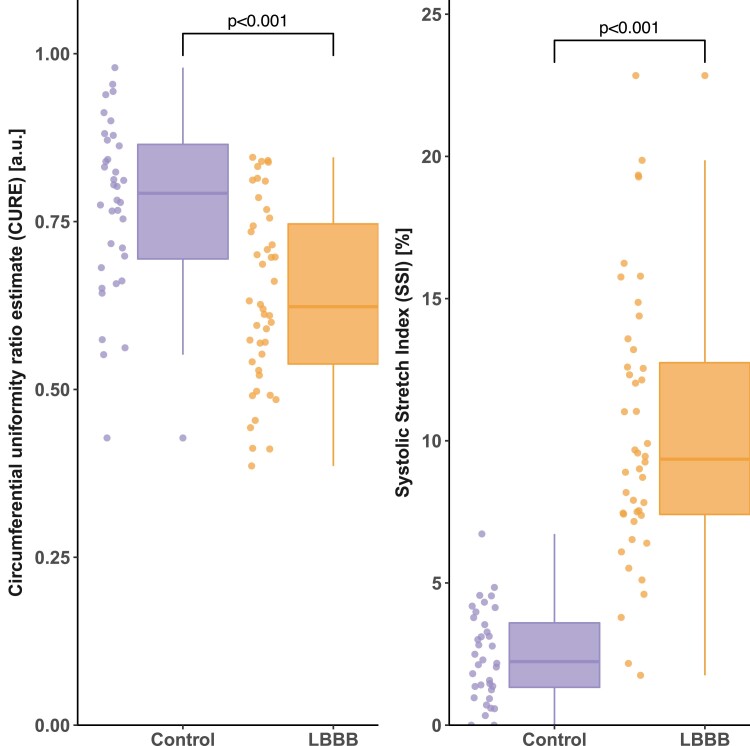
Values for CURE and SSI for LBBB and controls. The box and whisker plots show the median (horizontal line), interquartile range (box), and data points within 1.5× interquartile ranges of the first and third quartile, respectively (whiskers). Note there is more pronounced mechanical dyssynchrony (lower CURE and higher SSI) in LBBB compared with control. CURE is more homogenously distributed between groups compared with SSI.

**Figure 2:**
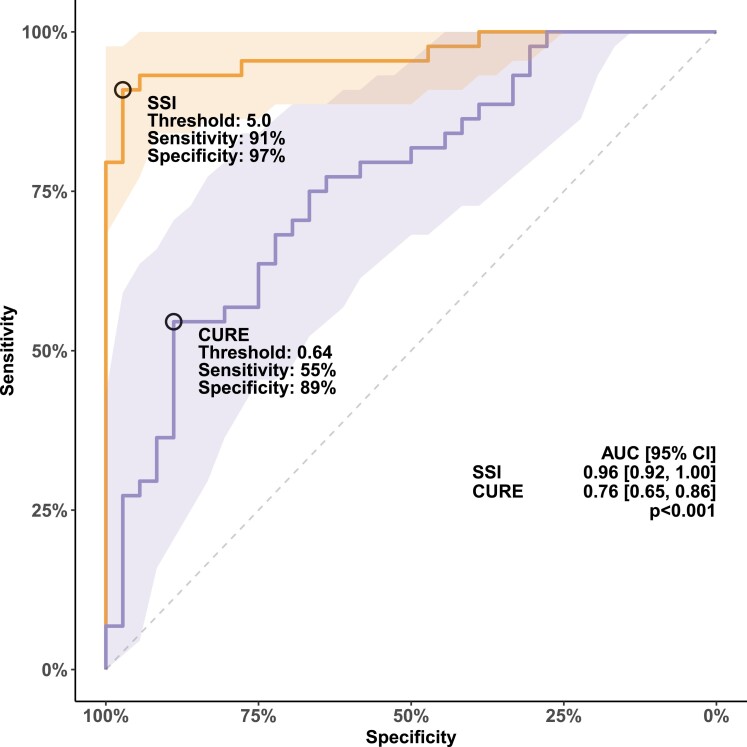
ROC curve for univariable logistic regression models to differentiate between LBBB and controls using CURE and SSI, respectively. Better discriminatory ability for LBBB is seen for SSI compared with CURE. Abbreviation: AUC, area under the ROC curve.

**Figure 3:**
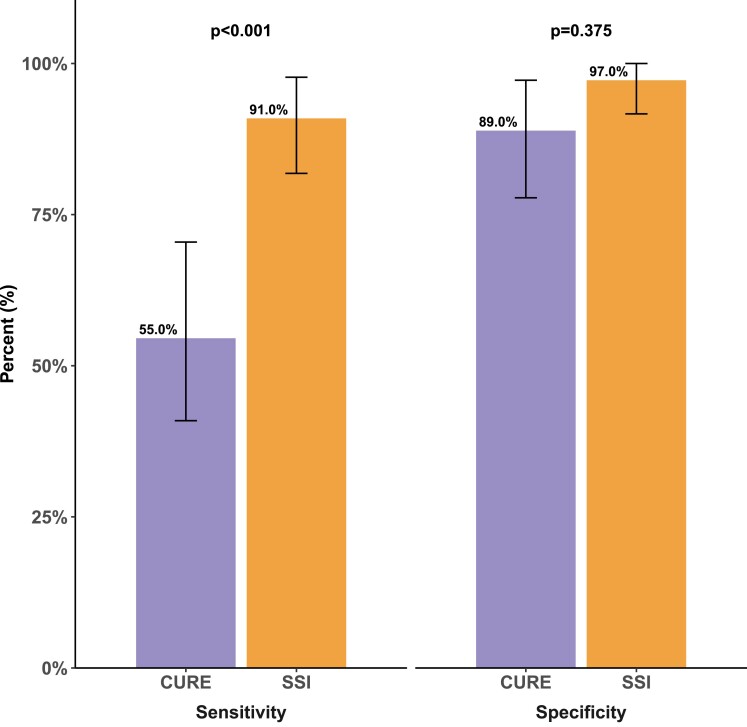
Bar plot showing sensitivity and specificity for detecting LBBB using SSI and CURE, respectively. Error bars denote 95% CIs.

Both CURE and SSI showed group differences between LBBB and controls (*Figure 1*). Consistent with a greater amount of mechanical dyssynchrony in LBBB, CURE was lower in LBBB compared with controls [0.62 (0.54–0.75) vs. 0.79 (0.69–0.86), *P* < 0.001], and SSI was higher in LBBB compared with controls [9.4 (7.4–12.7) vs. 2.2 (1.3–3.6), *P* < 0.001]. Compared with CURE, SSI had a greater area under the ROC curve for detecting mechanical dyssynchrony associated with strict LBBB [0.96 (0.92–1.00) vs. 0.76 (0.65–0.86), *P* < 0.001; *Figure 2*], and this corresponded to a higher sensitivity for SSI compared with CURE (*Figure 3*). The odds ratio for identifying LBBB for SSI was 2.77 (1.72–4.46) per 1% unit increase in SSI value and for CURE was 2.17 (1.45–3.26) per 0.10 decrease in CURE value. In evaluating the need for covariate-adjusted and/or covariate-specific ROC curves, linear regression models were used to test the association between dyssynchrony indices and covariates: age, LVEDVI, LVMI, and sex, allowing for interactions between LVMI and LVEDVI, with sex, respectively. No evidence was found in support of an association between dyssynchrony indices and covariates in controls or between CURE and covariates in LBBB. Furthermore, neither CURE nor SSI was associated with QRS duration in either LBBB or controls (P≥0.4 for all). There was an association between SSI and age, keeping other covariates fixed, in LBBB. This suggests that the discriminatory ability of SSI might vary with respect to age. For purposes of clarity, the unadjusted ROC curve is presented. Inter-rater and intra-rater ICCs for SSI were 0.86 (0.62–0.94) and 0.96 (0.91–0.98), respectively. Inter-rater and intra-rater ICCs for CURE were 0.77 (0.56–0.89) and 0.85 (0.70–0.93), respectively. Inter-rater and intra-rater SEMs for SSI were 1.26 (0.86–1.65) and 0.89 (0.60–1.15), respectively. Inter-rater and intra-rater SEMs for CURE were 0.07 (0.05–0.10) and 0.05 (0.03–0.06), respectively.

### CMR image availability

All CMR images analysed as part of the current study are made available online (doi:10.6084/m9.figshare.15155596). Among the individual CMR exams (*n* = 80), the majority were performed on a Siemens scanner (*n* = 75) and only a few on a Philips scanner (*n* = 5); all contain a cine short-axis stack, while most also contain cine two-chamber (*n* = 78), three-chamber (*n* = 79), and four-chamber images (*n* = 79). As per the inclusion criteria, all patients were verified to be free of myocardial scar by LGE CMR.

## Discussion

The main finding of the study is that the ability to discriminate between LBBB and QRS duration < 120 ms with normal QRS axis among patients with severely reduced LVEF and no scar was fair for CURE and excellent for SSI. This highlights that, when developing and evaluating indices aimed at accurately identifying mechanical dyssynchrony amenable to CRT, it is important to evaluate the performance of a proposed index both in patients with LBBB and in comparison with control subjects with QRS duration < 120 ms and normal QRS axis.

The lack of response and even harmful effects of CRT when implanted in patients with narrow QRS complexes indicates that CRT requires an electrical substrate.^25,26^ Findings that LBBB predicts greater benefit from CRT support that LBBB is that electrical substrate.^1–3^ Response to CRT can partly be explained by correction of the discoordinated contraction of myocardial wall segments, a consequence of abnormal electrical activation. However, not all patients with LBBB by conventional electrocardiographic criteria have complete LBBB,^27,28^ and mechanical dyssynchrony is not uniquely associated with electrical dyssynchrony.^29,30^ For example, focal LV myocardial scarring is also known to cause abnormalities in regional myocardial mechanics.^30^ Approximately one-third of patients with LBBB by conventional criteria do not have strict LBBB.^31^ Consequently, Strauss *et al*.^4^ proposed more strict criteria for LBBB. Increased rate of CRT response has been found when using this strict definition of LBBB,^5,6,32^ and strict LBBB is associated with greater mechanical dyssynchrony than non-strict LBBB.^33^ Mechanical dyssynchrony *per se* might therefore not be of primary interest in predicting CRT response but instead focus should be on identification and quantification of the mechanical dyssynchrony pattern associated with an abnormal electrical activation as seen in complete LBBB. Importantly, the current study evaluates the discriminatory ability of two recently proposed mechanical dyssynchrony indices for the mechanical dyssynchrony pattern associated with strict LBBB compared with patients with equally reduced ejection fraction but QRS duration < 120 ms and normal QRS axis, while controlling for confounding factors such as scar.

QRS duration cannot accurately characterize the spectrum of conduction abnormalities, and so it seems unlikely that any singular mechanical dyssynchrony index will be able to capture the full spectrum of variation inherent to dyssynchronous ventricular contraction. While both CURE and SSI capture differences in the pattern of discoordination on a group level, we found SSI superior to CURE with regard to the ability to differentiate between LBBB and controls. Our results suggest that the incremental mechanical dyssynchrony component associated with LBBB is better characterized by quantification of the absolute extent of stretch during the opposing directions of movement in the septum and lateral wall.

SSI was developed by Lumens *et al*.^18^ in an attempt to characterize the electromechanical substrate that may respond to CRT. They used a computational model to simulate electromechanical and non-electrical substrates of mechanical dyssynchrony and identified strain characteristics specific for the different substrates of mechanical dyssynchrony. In a study of patients enrolled in the Adaptive CRT trial, it was found that SSI by echocardiography was independently associated with CRT outcome, adjusting for QRS morphology, QRS duration, sex, heart failure aetiology, and treatment with angiotensin-converting enzyme inhibitors/angiotensin II receptor blockers.^17^ Whether SSI has added prognostic value over strict LBBB morphology is still unknown. We have found SSI able to accurately identify the electromechanical dyssynchrony pattern associated with strict LBBB. However, it remains to be explored whether SSI can be used to identify non-LBBB-wide QRS complex patients that may be suitable for CRT, and such studies are justified.

CURE was first evaluated in a canine model of heart failure and LBBB conduction delay.^14,15^ It was found that biventricular pacing leads to greater synchrony (increased CURE) and improved global function and that circumferential dyssynchrony indices had greater dynamic range when compared with longitudinal indices.^14^ Importantly, CURE was found sensitive to regionally clustered dyssynchrony.^14^ Regionally clustered dyssynchrony might show equal variance as dispersed dyssynchrony when compared with variance-based dyssynchrony measures, although with very different effects on cardiac mechanics.^14^ While CURE can be considered a more general measure of dyssynchrony, it has been found to be predictive of CRT response in clinical cohorts.^16,34,35^ An advantage of CURE over commonly used time-to-peak–based indices is that CURE utilizes information of the full cardiac cycle. Additionally, considering that CURE is derived from the relative positions of included segments and less so on their absolute value of strain, CURE is theoretically less sensitive to inter-vendor variations of strain measurements. However, despite these theoretical advantages of using CURE, the current study shows that CURE had a modest performance in identifying LBBB-specific mechanical dyssynchrony.

The association between mechanical dyssynchrony, quantified as the systolic dyssynchrony index, and myocardial scar has been studied in patients with systolic heart failure.^36^ They conclude that 25% of patients with narrow QRS (<130ms) presented with mechanical dyssynchrony, despite no difference in scar burden compared with narrow QRS patients without mechanical dyssynchrony. Those findings suggest that mechanical dyssynchrony in such patients might be secondary to myocardial scar rather than electrical dyssynchrony. There is no general agreement upon the definition of mechanical dyssynchrony, and the difference in vendor software for strain measurements limits straightforward comparisons between studies. CURE has been shown to identify a greater magnitude of dyssynchrony (lower CURE values) in patients with non-ischaemic cardiomyopathy compared with healthy controls (0.79±0.14 vs. 0.97±0.02).^37^ In a different study, CURE in healthy control volunteers has been shown to be 0.87±0.07.^38^ The current study shows that patients with severely reduced LVEF and QRS duration < 120 ms with normal QRS axis have some degree of mechanical dyssynchrony even in the absence of scar (median CURE 0.79). This would suggest that other factors beyond scar and LBBB contribute to mechanical dyssynchrony detected by CURE. Such factors may include variations in pre-load and/or afterload, and regional wall motion abnormalities due to chronic ischaemia or other non-ischaemic cardiomyopathies that impair contractile function without causing myocardial scar.

While both CURE and SSI displayed group differences between LBBB and controls, the current study found no evidence in support of a relationship between either CURE or SSI, and QRS duration within LBBB and control groups, respectively. CURE and QRS duration have previously been found to be modestly correlated (r=−0.58;P<0.001) in a cohort (n=43) of cardiomyopathy patients with similar reductions in ejection fraction and prolongation of QRS duration, although QRS morphology was not reported.^16^ However, when only evaluating the correlation between CURE and QRS duration in those patients referred for CRT (n=20), those authors found that the evidence did not support any correlation (r=−0.40;P=0.08).^16^ The apparent lack of correlation between dyssynchrony and QRS duration is of interest considering that current guidelines are still unclear regarding the group of patients with intermediate QRS width (QRS 120–149 ms). These exploratory results add to the notion that there is a complex relationship between electrical and mechanical dyssynchrony.

### Image availability

The current study shows that patients with severely reduced ejection fraction and QRS duration < 120 ms with normal QRS axis have a baseline level of mechanical dyssynchrony that is not attributable to myocardial scarring or prolonged depolarization of the myocardium. Consequently, when developing an index of mechanical dyssynchrony, specificity for its intended use should be of interest. To our knowledge, no study to date has included patients free of myocardial scar, with a QRS duration < 120 ms and normal QRS axis, and with severely reduced ejection fraction when comparing or developing indices of mechanical dyssynchrony. In order to facilitate future research where baseline mechanical dyssynchrony is accounted for, the images from the current study are made available online. See the section ‘Data Availability’ below.

### Limitations

Identification of the time point for aortic valve opening and aortic valve closure was performed by visual assessment of CMR cine images, and the accuracy of this assessment is limited by the temporal resolution of CMR images. However, any variations in accuracy would equally affect the analysis of both patient groups and hence should not have a major effect on the overall results. The current study performed feature-tracking strain analysis only in a single mid-ventricular SAX slice. However, this was due to increased through-plane motion in the basal slices and apical slices being more affected by partial volume effects, as well as septal dyssynchrony being visually less prominent towards the apex. The software used for strain analysis reports segmental strain measurements according to the American Heart Association 17-segment model. Hence, CURE calculation was limited to Fourier transformation applied to six individual myocardial segments in the mid-ventricular short-axis slice. The impact of the spatial resolution of measurement in CURE quantification has not previously been reported. However, it cannot be excluded that quantification of CURE using higher spatial resolution could potentially influence the results. The study included only patients without LV myocardial scar by CMR LGE; hence, accuracy for the two dyssynchrony indices in detecting LV mechanical dyssynchrony in the presence of scar could not be assessed. However, myocardial scar may impact the electromechanical association, is known to negatively affect strain measurements,^30^ and is negatively associated with CRT response.^16^ By only including patients free of LV myocardial scar and with LVEF ≤ 35%, the specific aim of the study was to increase specificity for detecting electromechanical dyssynchrony thought to be particularly amendable by CRT. The study compared subjects with LBBB by Strauss’ criteria to controls with QRS duration < 120 ms and QRS axis between −30° and +90°, not considering other potential CRT candidates such as those subjects with a non-LBBB QRS morphology but with a wide QRS (>130 ms) that according to guidelines receive a IIb (QRS > 150 ms receives a IIa) recommendation for CRT.^39^ However, LBBB QRS morphology has been found with a favourable CRT response compared with non-LBBB QRS morphology, and strict LBBB as per Strauss’ criteria has been found associated with a super-response to CRT.^6^ Since ECG criteria of LBBB QRS morphology by definition cannot be used for selecting subjects with non-LBBB QRS morphology, studying their mechanical dyssynchrony characteristics makes for a potentially promising starting point when trying to identify non-LBBB candidates for CRT.

## Conclusions

SSI was superior to CURE regarding the ability to discriminate between strict LBBB and QRS duration < 120 ms with normal QRS axis among patients with severely reduced ejection fraction and no scar. SSI merits further evaluation for detecting dyssynchrony in IVCD. The amount of dyssynchrony in patients with no scar, and severely reduced ejection fraction, needs to be taken into account when developing and evaluating indices aimed at accurately identifying mechanical dyssynchrony amenable to CRT. Such an evaluation should preferably include control subjects with QRS duration < 120 ms and normal QRS axis, severely reduced ejection fraction, and the absence of myocardial scar, and imaging data from such patients are provided for public use.

## Data Availability

The data sets generated and/or analysed during the current study, as well as the code needed to reproduce all aspects of the current study, are available in the Figshare repository (doi:10.6084/m9.figshare.15155596). The most recent version of the analysis code is available in the GitHub repository (https://github.com/dloewenstein/dillacs-study).

## References

[1] Zareba W, Klein H, Cygankiewicz I, Hall WJ, McNitt S, Brown M et al Effectiveness of cardiac resynchronization therapy by QRS morphology in the Multicenter Automatic Defibrillator Implantation Trial-Cardiac Resynchronization Therapy (MADIT-CRT). Circulation 2011;123:1061–72. 21357819 10.1161/CIRCULATIONAHA.110.960898

[2] Gold MR, Thebault C, Linde C, Abraham WT, Gerritse B, Ghio S et al Effect of QRS duration and morphology on cardiac resynchronization therapy outcomes in mild heart failure: results from the Resynchronization Reverses Remodeling in Systolic Left Ventricular Dysfunction (REVERSE) study. Circulation 2012;126:822–9. 22781424 10.1161/CIRCULATIONAHA.112.097709

[3] Bristow MR, Saxon LA, Boehmer J, Krueger S, Kass DA, De Marco T et al Cardiac-resynchronization therapy with or without an implantable defibrillator in advanced chronic heart failure. N Engl J Med 2004;350:2140–50. 15152059 10.1056/NEJMoa032423

[4] Strauss DG, Selvester RH, Wagner GS. Defining left bundle branch block in the era of cardiac resynchronization therapy. Am J Cardiol 2011;107:927–34. 21376930 10.1016/j.amjcard.2010.11.010

[5] Mascioli G, Padeletti L, Sassone B, Zecchin M, Lucca E, Sacchi S et al Electrocardiographic criteria of true left bundle branch block: a simple sign to predict a better clinical and instrumental response to CRT. Pacing Clin Electrophysiol 2012;35:927–34. 22651702 10.1111/j.1540-8159.2012.03427.x

[6] Tian Y, Zhang P, Li X, Gao Y, Zhu T, Wang L et al True complete left bundle branch block morphology strongly predicts good response to cardiac resynchronization therapy. Europace 2013;15:1499–506. 23468351 10.1093/europace/eut049

[7] Vereckei A, Katona G, Szelenyi Z, Szenasi G, Kozman B, Karadi I. The role of electrocardiography in the elaboration of a new paradigm in cardiac resynchronization therapy for patients with nonspecific intraventricular conduction disturbance. J Geriatr Cardiol 2016;13:118–25. 27168736 10.11909/j.issn.1671-5411.2016.02.002 PMC4854949

[8] Friedman DJ, Al-Khatib SM, Dalgaard F, Fudim M, Abraham WT, Cleland JGF et al Cardiac resynchronization therapy improves outcomes in patients with intraventricular conduction delay but not right bundle branch block: a patient-level meta-analysis of randomized controlled trials. Circulation 2023;147:812–23. 36700426 10.1161/CIRCULATIONAHA.122.062124 PMC10243743

[9] Rickard J, Michtalik H, Sharma R, Berger Z, Iyoha E, Green AR et al Predictors of response to cardiac resynchronization therapy: a systematic review. Int J Cardiol 2016;225:345–52. 27756040 10.1016/j.ijcard.2016.09.078

[10] Sipahi I, Chou JC, Hyden M, Rowland DY, Simon DI, Fang JC. Effect of QRS morphology on clinical event reduction with cardiac resynchronization therapy: meta-analysis of randomized controlled trials. Am Heart J 2012;163:260–7.e3. 22305845 10.1016/j.ahj.2011.11.014 PMC4113034

[11] Peichl P, Kautzner J, Čihák R, Bytešník J. The spectrum of inter- and intraventricular conduction abnormalities in patients eligible for cardiac resynchronization therapy. Pacing Clin Electrophysiol 2004;27:1105–12. 15305960 10.1111/j.1540-8159.2004.00592.x

[12] Morais P, Marchi A, Bogaert JA, Dresselaers T, Heyde B, D’hooge J et al Cardiovascular magnetic resonance myocardial feature tracking using a non-rigid, elastic image registration algorithm: assessment of variability in a real-life clinical setting. J Cardiovasc Magn Reson 2017;19:24. 28209163 10.1186/s12968-017-0333-y PMC5314711

[13] Heyde B, Jasaityte R, Barbosa D, Robesyn V, Bouchez S, Wouters P et al Elastic image registration versus speckle tracking for 2-D myocardial motion estimation: a direct comparison *in vivo*. IEEE Trans Med Imaging 2013;32:449–59. 23204281 10.1109/TMI.2012.2230114

[14] Helm RH, Leclercq C, Faris OP, Ozturk C, McVeigh E, Lardo AC et al Cardiac dyssynchrony analysis using circumferential versus longitudinal strain: implications for assessing cardiac resynchronization. Circulation 2005;111:2760–7. 15911694 10.1161/CIRCULATIONAHA.104.508457 PMC2396330

[15] Leclercq C, Faris O, Tunin R, Johnson J, Kato R, Evans F et al Systolic improvement and mechanical resynchronization does not require electrical synchrony in the dilated failing heart with left bundle-branch block. Circulation 2002;106:1760–3. 12356626 10.1161/01.cir.0000035037.11968.5c

[16] Bilchick KC, Dimaano V, Wu KC, Helm RH, Weiss RG, Lima JA et al Cardiac magnetic resonance assessment of dyssynchrony and myocardial scar predicts function class improvement following cardiac resynchronization therapy. JACC Cardiovasc Imaging 2008;1:561–8. 19356481 10.1016/j.jcmg.2008.04.013 PMC2678755

[17] Gorcsan J, Anderson CP, Tayal B, Sugahara M, Walmsley J, Starling RC et al Systolic stretch characterizes the electromechanical substrate responsive to cardiac resynchronization therapy. JACC Cardiovasc Imaging 2019;12:1741–52. 30219394 10.1016/j.jcmg.2018.07.013

[18] Lumens J, Tayal B, Walmsley J, Delgado-Montero A, Huntjens PR, Schwartzman D et al Differentiating electromechanical from non-electrical substrates of mechanical discoordination to identify responders to cardiac resynchronization therapy. Circ Cardiovasc Imaging 2015;8:e003744. 26338877 10.1161/CIRCIMAGING.115.003744

[19] R Core Team . R: A Language and Environment for Statistical Computing. Vienna, Austria: R Foundation for Statistical Computing; 2017.

[20] Wickham H, François R, Henry L, Müller K. *dplyr: A Grammar of Data Manipulation*. 2017.

[21] Wickham H . ggplot2: Elegant Graphics for Data Analysis. New York: Springer-Verlag New York; 2009.

[22] Robin X, Turck N, Hainard A, Tiberti N, Lisacek F, Sanchez J-C et al pROC: an open-source package for R and S+ to analyze and compare ROC curves. BMC bioinformatics 2011;12:77. 21414208 10.1186/1471-2105-12-77 PMC3068975

[23] Caldwell AR . SimplyAgree: an R package and jamovi module for simplifying agreement and reliability analyses. J Open Source Softw 2022;7:4148.

[24] Xie Y. *knitr: A General-Purpose Package for Dynamic Report Generation in R*. 2017.

[25] Beshai JF, Grimm RA, Nagueh SF, Baker JH, Beau SL, Greenberg SM et al Cardiac-resynchronization therapy in heart failure with narrow QRS complexes. N Engl J Med 2007;357:2461–71. 17986493 10.1056/NEJMoa0706695

[26] Ruschitzka F, Abraham WT, Singh JP, Bax JJ, Borer JS, Brugada J et al Cardiac-resynchronization therapy in heart failure with a narrow QRS complex. N Engl J Med 2013;369:1395–405. 23998714 10.1056/NEJMoa1306687

[27] Vassallo JA, Cassidy DM, Marchlinski FE, Buxton AE, Waxman HL, Doherty JU et al Endocardial activation of left bundle branch block. Circulation 1984;69:914–23. 6705167 10.1161/01.cir.69.5.914

[28] Auricchio A, Fantoni C, Regoli F, Carbucicchio C, Goette A, Geller C et al Characterization of left ventricular activation in patients with heart failure and left bundle-branch block. Circulation 2004;109:1133–9. 14993135 10.1161/01.CIR.0000118502.91105.F6

[29] Jackson T, Amraoui S, Sohal M, Sammut E, Behar JM, Claridge S et al The interaction of QRS duration with cardiac magnetic resonance derived scar and mechanical dyssynchrony in systolic heart failure: implications for cardiac resynchronization therapy. IJC Heart Vasc 2018;18:81–5. 10.1016/j.ijcha.2017.11.005 PMC5941225 29750182

[30] Maret E, Todt T, Brudin L, Nylander E, Swahn E, Ohlsson JL et al Functional measurements based on feature tracking of cine magnetic resonance images identify left ventricular segments with myocardial scar. Cardiovasc Ultrasound 2009;7:53. 19917130 10.1186/1476-7120-7-53 PMC2785780

[31] Strauss DG . Differentiation between left bundle branch block and left ventricular hypertrophy: implications for cardiac resynchronization therapy. J Electrocardiol 2012;45:635–9. 23022304 10.1016/j.jelectrocard.2012.09.001

[32] Jastrzębski M, Kukla P, Kisiel R, Fijorek K, Moskal P, Czarnecka D. Comparison of four LBBB definitions for predicting mortality in patients receiving cardiac resynchronization therapy. Ann Noninvasive Electrocardiol 2018;23:e12563. 29806716 10.1111/anec.12563 PMC6931883

[33] Andersson LG, Wu KC, Wieslander B, Loring Z, Frank TF, Maynard C et al Left ventricular mechanical dyssynchrony by cardiac magnetic resonance is greater in patients with strict vs nonstrict electrocardiogram criteria for left bundle-branch block. Am Heart J 2013;165:956–63. 23708167 10.1016/j.ahj.2013.03.013 PMC3664936

[34] Bilchick KC, Kuruvilla S, Hamirani YS, Ramachandran R, Clarke SA, Parker KM. Impact of mechanical activation, scar, and electrical timing on cardiac resynchronization therapy response and clinical outcomes. J Am Coll Cardiol 2014;63:1657–66. 24583155 10.1016/j.jacc.2014.02.533 PMC4427624

[35] Ramachandran R, Chen X, Kramer CM, Epstein FH, Bilchick KC. Singular value decomposition applied to cardiac strain from MR imaging for selection of optimal cardiac resynchronization therapy candidates. Radiology 2015;275:413–20. 25581423 10.1148/radiol.14141578 PMC4456179

[36] Jackson T, Claridge S, Behar J, Sammut E, Webb J, Carr-White G et al Narrow QRS systolic heart failure: is there a target for cardiac resynchronization? Expert Rev Cardiovasc Ther 2015;13:783–97. 26048215 10.1586/14779072.2015.1049945

[37] Taylor RJ, Umar F, Moody WE, Meyyappan C, Stegemann B, Townend JN et al Feature-tracking cardiovascular magnetic resonance as a novel technique for the assessment of mechanical dyssynchrony. Int J Cardiol 2014;175:120–5. 24852836 10.1016/j.ijcard.2014.04.268

[38] Kowallick JT, Morton G, Lamata P, Jogiya R, Kutty S, Hasenfuß G et al Quantitative assessment of left ventricular mechanical dyssynchrony using cine cardiovascular magnetic resonance imaging: inter-study reproducibility. JRSM Cardiovasc Dis 2017;6:2048004017710142. 28567282 10.1177/2048004017710142 PMC5438106

[39] Glikson M, Nielsen JC, Kronborg MB, Michowitz Y, Auricchio A, Barbash IM et al 2021 ESC guidelines on cardiac pacing and cardiac resynchronization therapy: developed by the task force on cardiac pacing and cardiac resynchronization therapy of the European Society of Cardiology (ESC) with the special contribution of the European Heart Rhythm Association (EHRA). Eur Heart J 2021;42:3427–520.34455430

